# Multiple Adjacent Isolated Thoracic Spinous Process Fractures in High-Energy Trauma

**DOI:** 10.1155/2015/921526

**Published:** 2015-06-17

**Authors:** Jacob M. Kirsch, Amit Nathani, Rakesh D. Patel

**Affiliations:** Department of Orthopaedic Surgery, University of Michigan, Ann Arbor, MI 48109, USA

## Abstract

Isolated thoracic spinous process fractures involving multiple adjacent vertebral segments are a rare occurrence in the setting of high-energy trauma. These findings should prompt further investigation to exclude other concomitant osseous or ligamentous injuries. Evaluation by computed tomography is often most useful to detect these fractures. Proper treatment of extensive multilevel injury is poorly defined in the literature. In our experience, conservative management consisting of initial bracing with graduated lifting restrictions has produced excellent functional results.

## 1. Introduction

The term “Clay-Shoveler's fracture” was originally coined in 1940 to describe isolated spinous process fractures occurring from C6-T3 in Western Australian laborers [1]. In the modern era, this fracture pattern has been observed in a variety of patients, including football players, power-lifters, golfers, and trauma patients [2–6]. There have been only three previous reports in the literature documenting isolated thoracic spinous process fractures involving five or more contiguous vertebrae [2, 5, 7]. Despite the paucity of published literature, this entity may be more common than previously believed. We report two cases that presented to our institution over the past year of contiguous isolated thoracic spinous process fractures occurring in the setting of high-energy trauma.

## 2. Case Discussion

### 2.1. Patient 1

**Figure 1:**
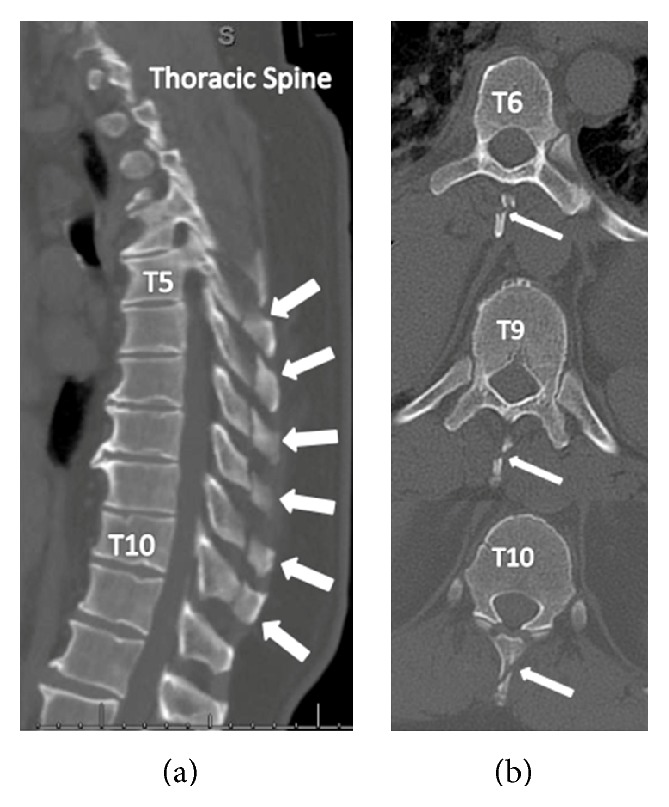
(a) Sagittal computed tomography (CT) image demonstrating thoracic spinous process fractures (arrows) spanning from T5 to T10. (b) Axial CT showing select (T6, T9, and T10) spinous process fractures (arrows). No evidence of vertebral body or intralaminar extension is noted.

**Figure 2:**
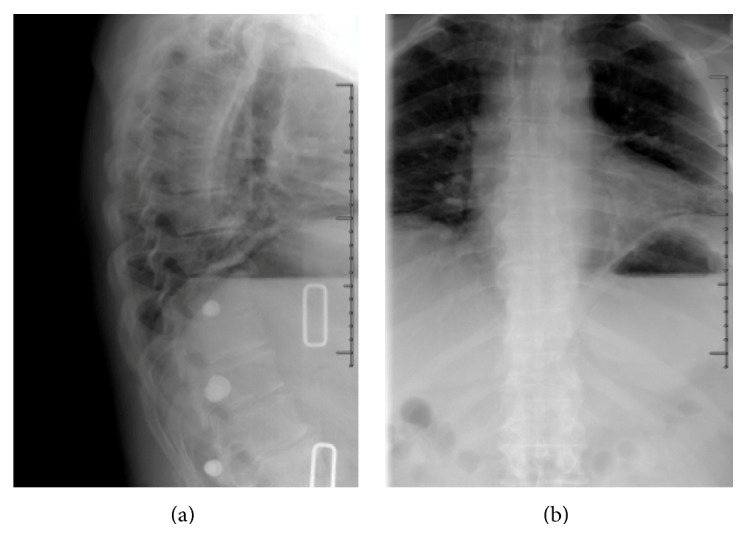
Sagittal (a) and anteroposterior (b) plain film in thoracolumbar sacral orthosis brace at 10 weeks after the initial injury, demonstrating good osseous union and well-maintained sagittal alignment.

A 53-year-old male was involved in a motorcycle collision at a speed of approximately 40 mph. Physical exam was significant for right-sided paraspinal tenderness in the thoracic region. He had no neurologic deficits. Computed tomography (CT) revealed thoracic spinous process fractures within T5–T10 without evidence of vertebral body injury, extension into the lamina, or ligamentous compromise (Figure 1). He also sustained right-sided posterolateral rib fractures involving ribs 3, 4, 6, and 7. The patient was initially placed in a thoracolumbar sacral orthosis (TLSO) brace with 5-pound lifting restrictions for 6 weeks; after which time the brace was discontinued and he was increased to 25-pound lifting restrictions. The patient was completely asymptomatic with well-maintained spinal alignment 10 weeks after his injury (Figure 2). At that point, he was allowed to return to full activities without any restriction.

### 2.2. Patient 2

**Figure 3:**
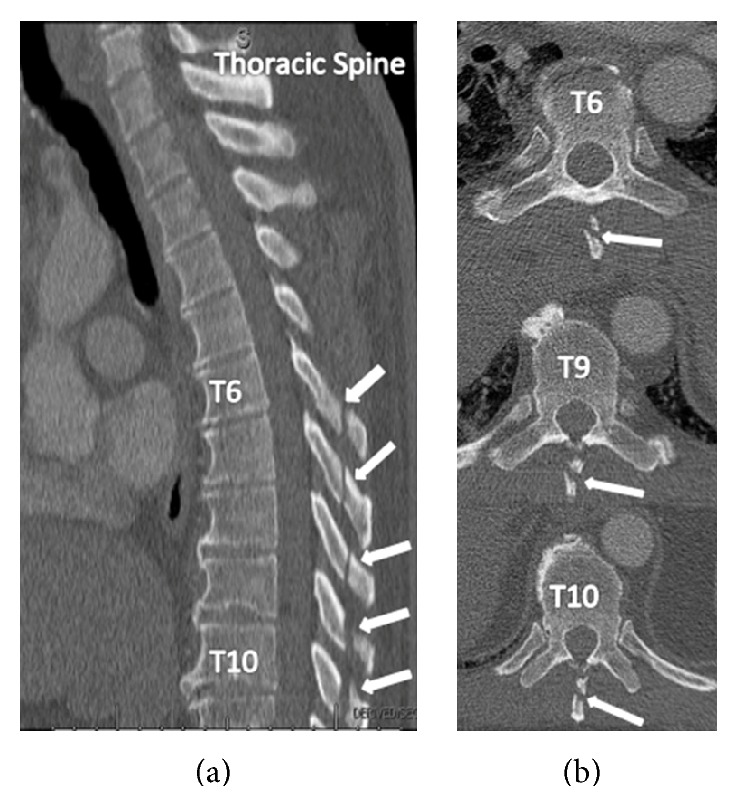
(a) Sagittal CT demonstrating thoracic spinous process fractures (arrows) spanning from T6 to T10. (b) Axial CT showing select (T6, T9, and T10) spinous process fractures (arrows). No evidence of vertebral body or intralaminar extension is noted.

A 53-year-old man without a helmet lost control of his motorcycle and collided with a guardrail. He had loss of consciousness and an initial Glasgow coma scale score of 12. Physical exam revealed no gross abnormalities of the spine; however he expressed significant left-sided paraspinal tenderness in the thoracic region. CT of his spine revealed isolated fractures of the thoracic spinous processes within T6–T10 without any other evidence of spine pathology (Figure 3). He also sustained fractures of ribs 4–8 on the left side. The patient was placed in a TLSO brace and treated in a similar fashion as described previously.

## 3. Discussion

Isolated thoracic spinous process fractures involving five or more contiguous vertebrae are rarely encountered in the orthopaedic literature. These fractures often occur in conjunction with other bony or soft tissue injuries of the vertebral column. The two aforementioned cases almost double the reported literature on this specific fracture pattern. Although this pattern of injury has been referred to elsewhere as “Clay-Shoveler's fracture,” it should be considered a different entity based on the energy mechanism, level, and extent of contiguous segment involvement. Meyer et al. [8] described several mechanisms associated with spinous process fractures of the lower cervical and upper thoracic region. In patients with multiple adjacent isolated thoracic spinous process fractures associated with high-energy trauma, the mechanism of injury is likely a combination of hyperflexion/hyperextension and direct trauma [2, 5]. This mechanism is distinct from the simple avulsion-type injury originally described as a “Clay-Shoveler's fracture.”

Other authors have recently reported cases of extensive multilevel spinous process fractures involving the cervicothoracic junction [9, 10]. However, the cervicothoracic junction represents a unique transition point between a more mobile cervical spine and a relatively rigid thoracic spine. As a result of the intrinsic biomechanical properties of the spine at this particular region, there is increased stress, which predisposes it to injury [11]. Conversely, in the two patients presented above, the region of injury was limited to the thoracic spine, thereby precluding the biomechanical forces acting at the cervicothoracic junction.

Isolated thoracic spinous process fractures are stable injuries, which are routinely treated with conservative therapy. Even in the setting of multilevel contiguous fractures, excellent functional outcomes are commonplace with nonoperative therapy. Conservative treatment with a TLSO brace for 6 weeks with graduated lifting restrictions for up to 10 weeks has produced great results in our experience, with both patients going on to pain-free full recovery. Persistent and focal pain over the spinous processes beyond this time period should raise concern for a symptomatic pseudoarthrosis. It is well documented that spinous process fractures infrequently achieve osseous union [12]; however, the vast majority are asymptomatic. Evaluation of a suspected symptomatic pseudoarthrosis is most reliable with CT imaging. Other imaging modalities such as magnetic resonance imaging (MRI) and technetium bone scan with single-photon emission computed tomography (SPECT) can be used but are of more limited value [13]. Surgical excision of the spinous process fragment is the treatment of choice in these symptomatic patients who are refractory to nonoperative management.

Isolated thoracic spinous process fractures in the setting of high-energy trauma are a rare occurrence. Involvement of five or more contiguous thoracic vertebral segments has been reported only three times previously in the literature [2, 5, 7]. In the acute setting, particularly following high-energy trauma, it is imperative to rule out other osseous and ligamentous damage to ensure spinal column stability before commencing with conservative treatment.
